# Irradiation-Induced Defect Engineering in REBCO Coated Conductors: Mechanisms, Effects, and Perspectives

**DOI:** 10.3390/ma19020300

**Published:** 2026-01-12

**Authors:** Yuxiang Li, Ningning Liu, Ziheng Guo, Liangkang Chen, Dongliang Gong, Dongliang Wang, Yanwei Ma

**Affiliations:** 1 School of Material Science and Engineering, University of Zhengzhou, Zhengzhou 450001, China; liyx@mail.iee.ac.cn; 2 Key Laboratory of Applied Superconductivity, Institute of Electrical Engineering, Chinese Academy of Sciences, Beijing 100190, China; liuningning@mail.iee.ac.cn (N.L.); guoziheng@mail.iee.ac.cn (Z.G.); chenliangkang@mail.iee.ac.cn (L.C.); ywma@mail.iee.ac.cn (Y.M.); 3 University of Chinese Academy of Sciences, Beijing 100049, China

**Keywords:** REBCO coated conductors, irradiation-induced defects, flux pinning, proton irradiation, heavy-ion irradiation, mixed irradiation, superconducting performance

## Abstract

REBa_2_Cu_3_O_7−*δ*_ (REBCO) coated conductors are considered a critical material for next-generation high-field superconducting applications owing to their superior superconducting performance at elevated temperatures and under strong magnetic fields. However, rapid degradation of the critical current density ($J_{c}$) under high-field and high-temperature conditions remains a major limitation for their practical applications. To address this, controlling flux pinning centers has emerged as a crucial strategy to enhance performance. Irradiation techniques, as one of the most commonly employed methods, have attracted considerable attention due to their capability to provide precise control, high reproducibility, and flexibility in tailoring the microstructure. In this review, we focus on the effects of proton, heavy-ion, and neutron irradiation on the microstructure and superconducting properties of REBCO coated conductors. We discuss the underlying mechanisms in terms of defect types and distributions, energy loss processes, flux pinning enhancement, and the evolution of $J_{c}$ and transition temperature ($T_{c}$). Furthermore, we compare different irradiation methods, highlighting their advantages and suitability across diverse temperature and magnetic field conditions. The potential of hybrid irradiation strategies for creating multiscale composite pinning landscapes is also examined. Future efforts should aim to synergistically combine different irradiation mechanisms and optimize defect structures to develop REBCO tapes with highly isotropic and stable flux pinning, which is essential for large-scale applications in fusion energy, high-field magnets, and aerospace electric motors.

## 1. Introduction

REBa_2_Cu_3_O_7−*δ*_ (REBCO, where RE represents Y, Gd, Sm or other rare earth elements) coated conductors based on biaxial texture are considered ideal candidate materials for high-field applications due to their excellent critical magnetic field and current-carrying capacity at elevated temperatures [1,2]. They have already been deployed in multiple demonstration projects, including power transmission grids, fusion magnets, smart grids, MRI systems, and high-field scientific equipment [3]. However, the inherent deficiency in flux pinning centers leads to a dramatic degradation of critical current density ($J_{c}$) under high magnetic fields (defined here as the thin-film regime of several tesla, typically 5–10 T), severely limiting their applications in extreme environments such as fusion reactors and high-field magnets [4,5]. This primarily results from the low density of natural defects in REBCO films, where well-bonded grain boundaries coexist with a lack of strongly ordered artificial pinning centers, resulting in insufficient suppression of the motion of flux vortices driven by Lorentz forces [6,7]. Since flux lines can be suppressed by crystal defects and impurities in the material, researchers have adopted the introduction of various types of defects as flux pinning centers (PCs) as a crucial strategy for enhancing $J_{c}$ [8,9].

Over the past few decades, researchers have attempted to incorporate non-superconducting secondary phases such as BaHfO_3_, BaZrO_3_, and BaSnO_3_ to create effective pinning centers in REBCO thin films. However, precise control of these secondary phase defects remains challenging due to their introduction during the growth process [10,11]. Therefore, irradiation has garnered significant attention in recent years as a controllable and effective method for material modification in superconducting research. In the development of REBCO coated conductors, irradiation techniques have been employed to precisely tailor their microstructure, particularly by controlling ion type, energy, and irradiation angle to generate point defects, columnar defects, and other defect structures with well-regulated size, density, and distribution. This approach has proven highly effective in optimizing flux pinning properties and enhancing critical current density [12,13,14].

The advantages of irradiation modulation are primarily reflected in its high controllability, efficiency, and flexibility [15]. The irradiation parameters, including particle type, energy, and fluence, can be precisely tuned to enable fine control over the density and spatial distribution of defects. Compared to conventional heat treatment or chemical doping methods, irradiation offers superior reproducibility and controllability in defect generation [16,17]. Different irradiation particles (e.g., protons, heavy ions, neutrons) can generate distinct defect types, endowing this technique with exceptional versatility and adaptability for superconducting material optimization [18,19,20]. Moreover, irradiation can be employed to tailor material properties under various temperature and magnetic field conditions, and can also be combined with other physical modulation methods, such as annealing and mechanical stress [21,22]. Due to the diverse types of pinning defects introduced by various irradiation methods, their mechanisms of action and effective ranges differ significantly. This review primarily focuses on the effects of several major irradiation types, such as proton, heavy ion, and neutron irradiation, on the properties and performance of superconducting thin film materials. We further discuss the underlying mechanisms responsible for these effects and highlight the temperature and magnetic field regimes where substantial performance enhancements are observed, thereby providing a foundation for further improvement and application of second-generation high-temperature superconducting films.

## 2. Fundamentals of Irradiation Physics

### 2.1. Electronic Energy Loss (EEL) and Nuclear Energy Loss (NEL)

**Figure 1:**
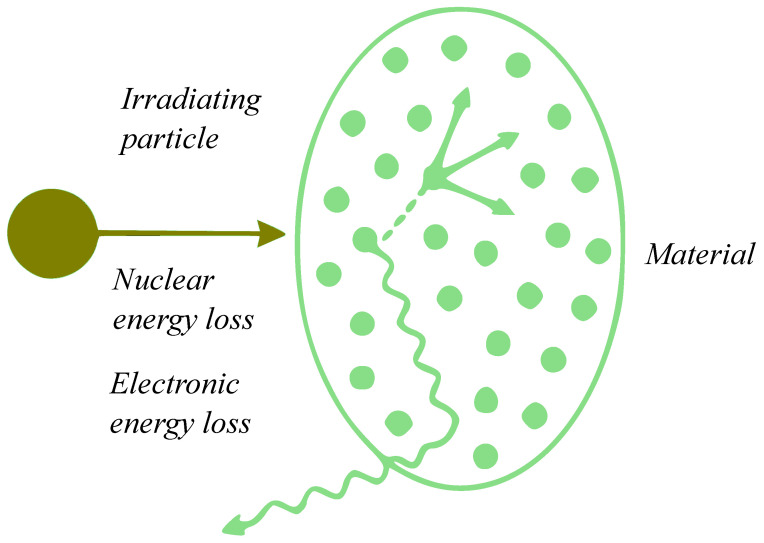
Schematic illustration of NEL and EEL during irradiation.

During irradiation, incident ions interact with material atoms and transfer their energy to the material. Such interactions can be categorized into two main types: electronic energy loss (Electronic Energy Loss, EEL) and nuclear energy loss (Nuclear Energy Loss, NEL) [23] (Figure 1). These two energy loss mechanisms play a crucial role in defect formation within the material and directly influence the irradiation-induced damage characteristics and performance changes of the material [24].

EEL refers to the energy lost by incident ions per unit length as they pass through a material, primarily through interactions with the electrons in the target material, such as ionizing and exciting target electrons [25]. This energy loss mainly occurs via the electric field effect, causing collisions between the irradiating ions and the electrons in the material [26]. Furthermore, the generation of EEL not only leads to excitation of electrons in the material but may also induce low-energy excited states or chemical reactions, which can create defects in the microstructure of the material. Generally, EEL significantly influences irradiation damage in materials, especially under high-energy irradiation, where it plays a dominant role in the initial damage mechanisms [27].

NEL refers to the process in which irradiating particles collide with atomic nuclei in the material, transferring a portion of their kinetic energy to the atoms and causing atomic displacements [24]. Compared to EEL, NEL has a more pronounced impact on the atomic structure of the material, especially during interactions with low−energy irradiating particles [26].

### 2.2. SRIM and dpa Calculation Methods

EEL and NEL exist simultaneously during irradiation and interact and jointly determine the irradiation damage characteristics of materials [23]. The energy transferred by irradiating particles in the material is mainly divided into two parts: one part excites electrons (EEL), while the other part is transferred to target atoms, causing their displacement (NEL). In high-energy irradiation, EEL typically dominates, leading to a large number of electronic excitations and excited states in the material. These excited states may induce defect formation through chemical reactions or changes in atomic interactions [28]. On the other hand, NEL causes displacement damage and lattice defects through collisions with atomic nuclei, which is particularly significant in low-energy irradiation. By controlling the type, energy, and fluence of irradiating particles, the ratio of electronic to NEL can be precisely adjusted to modulate the irradiation damage characteristics and final performance of the material [29].

Due to the complex interplay of EEL and NEL during irradiation, which is difficult to directly observe experimentally, researchers commonly use Stopping and Range of Ions in Matter (SRIM) simulations to more accurately predict irradiation outcomes. SRIM is a widely applied theoretical tool for studying irradiation processes, capable of simulating the propagation paths, energy losses, and interactions of irradiating particles within materials. The model is based on the physical principles of electronic and NEL, calculating the trajectories and interactions of particles in the material to predict defect generation [23].

The degree of irradiation damage induced by low-energy ions can be quantified by the ratio of the total number of displaced atoms per unit volume to the atomic density, expressed as Displacements Per Atom (dpa). This parameter represents the average number of times lattice atoms in a solid are displaced due to irradiation at a given dose [30]. If the incident ion species, energy, and the target material’s chemical composition, density, and displacement threshold energy are known, the Transport of Ions in Matter (TRIM) module within the SRIM Monte Carlo program can be used to calculate the number of displacements and ion penetration depth in the material after low-energy ion irradiation. This enables further calculation of the material’s dpa value, specifically as follows:

(1) $$\mathrm{dpa}=\frac{A}{\rho N_{A}d}N_{F}\Phi ,$$

Here, *A* is the molar mass of the sample (in g/mol), *d* is the ion penetration depth provided by the SRIM/TRIM simulation, ϕ is the total number of incident particles passing through a unit area, ρ is the material density, N_*A*_ is Avogadro’s constant, and N_*F*_ represents the number of Frenkel pairs, which is obtained from SRIM/TRIM simulations using the Full Cascade calculation displacement model [31].

## 3. Proton Irradiation

### 3.1. Types of Defects Induced

Proton irradiation generates various types of defects through interactions with the material, which play crucial roles in superconductors, particularly in enhancing flux pinning. The primary defects introduced by proton irradiation include point defects and small-size defect clusters [32,33,34]. These defects suppress the motion of magnetic vortices, thereby enhancing the flux pinning capability of the superconducting material and improving its performance under low temperatures and high magnetic fields.

Point defects, such as vacancies and interstitials, are the most fundamental types of defects formed during proton irradiation. These defects are produced when protons collide with atomic nuclei in the material, transferring kinetic energy to target atoms and displacing them from their original lattice positions. This process creates vacancies. Simultaneously, some atoms are pushed into interstitial sites, forming interstitial defects. Such point defects result in localized lattice distortions that serve as effective flux pinning centers [35]. In superconducting materials, vacancies can attract magnetic flux lines, while interstitials can modify the local electronic structure of the lattice, increasing electron scattering and thus enhancing the critical current density ($J_{c}$). By adjusting the irradiation fluence, the density of point defects can be tuned, allowing for the optimization of superconducting properties.

**Figure 2:**
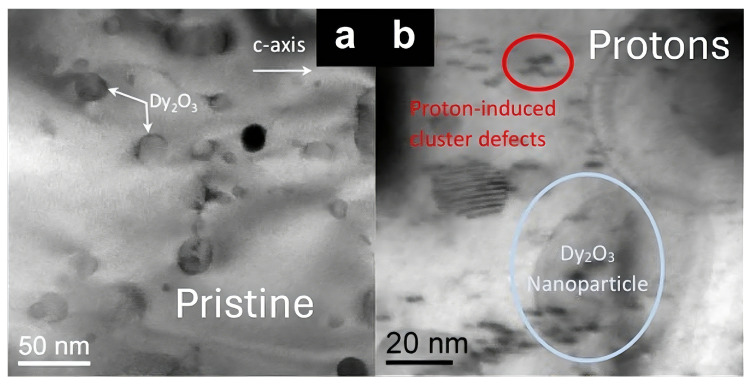
Transmission electron microscopy images of YBCO coated conductors fabricated by metal–organic deposition (MOD): (**a**) pristine sample, showing pre-existing Dy_2_O_3_ nanoparticles within the REBCO matrix; (**b**) sample after irradiation with 4 MeV protons to a fluence of $1.8\times 10^{17}$ cm^−2^, revealing additional nanoscale proton-induced defect clusters superimposed on the original microstructure [36].

Transmission electron microscopy provides direct microstructural evidence for the defect landscape introduced by proton irradiation in YBCO coated conductors. As shown in Figure 2, the pristine sample exhibits a relatively homogeneous microstructure, in which pre-existing secondary phase nanoparticles such as Dy_2_O_3_ are embedded in an otherwise well-ordered REBCO matrix. After irradiation with 4 MeV protons to a fluence of $1.8\times 10^{17}$ cm^−2^, additional nanoscale contrast features become visible, which are absent in the pristine conductor. These irradiation-induced features appear as small, diffuse defect clusters distributed throughout the superconducting layer, indicating the formation of proton-induced defect aggregates superimposed on the original nanoparticle landscape.

Proton irradiation primarily generates point defects and small defect clusters through nuclear energy loss processes, which are characterized by relatively low migration energy barriers. As a result, these defects are more susceptible to thermally activated recovery during annealing or repeated thermal cycling. Experimental studies have shown that post-irradiation annealing can lead to a partial restoration of the superconducting transition temperature, indicating the recombination or rearrangement of irradiation-induced point defects [37]. However, such thermal recovery is often accompanied by a reduction in the irradiation-enhanced flux pinning contribution, reflecting the metastable nature of proton-induced defect structures. Therefore, while proton irradiation provides an effective means to enhance isotropic background pinning, its long-term stability is strongly influenced by subsequent thermal treatments and operating temperature conditions.

### 3.2. Mechanism of Defect-Induced Pinning Forces

During proton irradiation, the defect type, density, and size collectively determine the strength and effectiveness of the pinning mechanism [38]. When irradiated with low-energy protons, atomic displacements occur, leading to the formation of Frenkel defects (i.e., vacancy–interstitial pairs). As incident protons collide with atoms in the YBCO lattice, NEL dominates, displacing light oxygen atoms from their lattice sites and forming oxygen vacancies and interstitial oxygen atoms. Due to the low mass and energy of protons, the resulting damage mainly consists of point defects [32]. Therefore, in the case of REBCO, the pinning force primarily originates from the weak pinning caused by copper and oxygen point defects generated by proton irradiation [39]. M.L. Griffith et al. [39] conducted annealing experiments on samples with significantly enhanced $j_{c}$ after low-fluence irradiation. The results showed that as copper and oxygen atoms recombined, the enhanced $j_{c}$ gradually returned to its original level. Subsequent studies using Raman spectroscopy further analyzed samples subjected to various proton irradiation fluences and provided deeper insights into the mechanism of point defect formation induced by proton irradiation [40].

**Figure 3:**
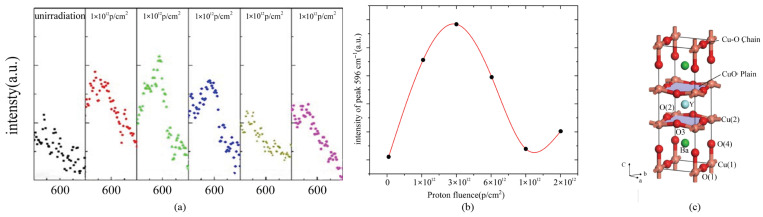
(**a**,**b**) Raman peak intensity at 596 cm^−1^ as a function of irradiation fluence [40]. (**c**) Crystal structure of YBCO [42].

Figure 3 depicts the variation of the characteristic Raman peak intensity at 596 cm^−1^ of YBCO samples after irradiation as a function of irradiation fluence. This vibrational mode is attributed to the apical oxygen O1 [41]. With increasing irradiation fluence, the characteristic peak intensity increases due to Cu–O chain breakage. Subsequently, excess O2 atoms and interstitial oxygen atoms may migrate to the O1 site, leading to the repair of broken Cu–O bonds and causing a slight decrease in the 596 cm^−1^ peak intensity. Raman measurements thus indicate that the primary defect component induced by proton irradiation is the displacement of oxygen atoms in the Cu–O chains, and these oxygen defects act as effective flux vortex pinning centers, enhancing the critical current density of REBCO at specific temperatures.

The weak pinning centers introduced by proton irradiation are relatively small in size and located within the normal regions of the superconductor. These pinning centers are numerous and have a small volume, effectively restricting the motion of small-scale magnetic flux. At low temperatures (<10 K) and high magnetic fields, due to the relatively small vortex size and weak thermal fluctuations, point defects serve as the dominant pinning centers. Their size is approximately $\xi^{3}$ and the temperature dependence of the effectiveness of weak pinning centers has been shown to approximately follow an exponential form [43].

(2) $$J_{c}^{\mathrm{WK}}\left(T\right)=J_{c}^{\mathrm{WK}}\left(0\right)\exp\left(-\frac{T}{T_{-}}\right),$$

$J_{c}^{\mathrm{WK}}$ is the contribution of weak pinning centers at 0 K.

At low temperatures and high magnetic fields, weak point pinning centers can significantly enhance flux pinning, preventing flux creep and thereby preserving the current-carrying capability of the superconductor. Small-sized defects generated by proton irradiation act as these weak pinning centers, leading to an increase in the normal-state resistivity [34]. Meanwhile, an appropriate amount of vortex pinning induced by point defects reduces the activation energy $U_{0}\left(H\right)$ in the low magnetic field region, while causing a gradual decrease in $U_{0}\left(H\right)$ in the high magnetic field region [44].

### 3.3. Effect of Proton Irradiation on REBCO Properties

The defect density and size introduced by proton irradiation are critical factors influencing the superconducting critical current density ($J_{c}$). As the proton irradiation fluence increases, defect density also rises, effectively enhancing flux pinning and improving the critical current density of the material. However, excessive defect density may lead to adverse effects such as structural degradation and lattice distortion, which can result in the deterioration of superconducting performance.

Defect size also plays a crucial role. At relatively low irradiation fluence, the generated small-sized defects can effectively pin magnetic flux and enhance the critical current density ($J_{c}$) of the superconductor. When the defect size matches the coherence length, the pinning force reaches its maximum [44]. These small defects can form weak or small-scale pinning centers that effectively restrict vortex motion under low temperatures and high magnetic fields. However, as the irradiation fluence continues to increase, defect size also grows. Especially under high-fluence proton irradiation, large defects may no longer enhance flux pinning and may even suppress the superconducting properties.

At appropriate fluence, small point defects are typically capable of effectively pinning magnetic vortices at low temperatures, thereby enhancing the critical current density ($J_{c}$) of the superconductor. These point defects, by forming weak or small-scale pinning centers, effectively restrict vortex motion under low-temperature and high-field conditions [45]. As temperature increases, however, individual point pinning becomes insufficient to restrain vortex motion, leading to a significant decrease in pinning strength. Nonetheless, since proton irradiation is usually applied on top of the material’s intrinsic defects, point defects generated at optimal fluence can cooperate with intrinsic pinning centers to form a mixed pinning landscape, resulting in enhanced vortex immobilization. This synergistic effect is particularly effective under low-temperature and high-field conditions, where proton irradiation leads to a more pronounced increase in $J_{c}$ [32,34].

**Figure 4:**
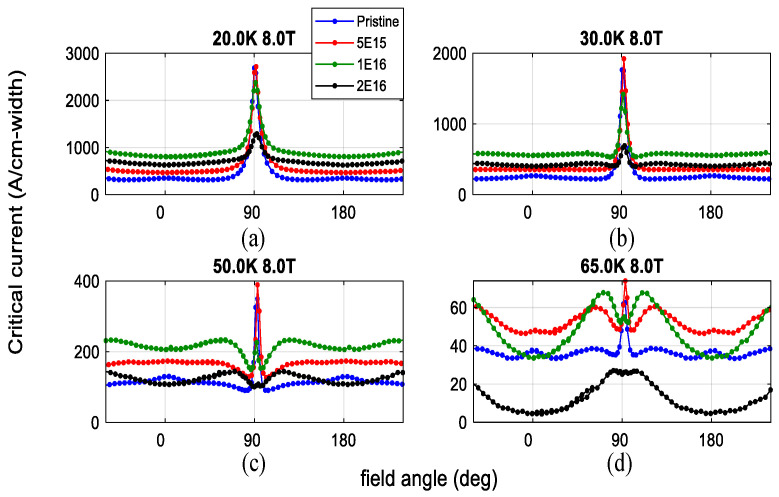
Angular dependence of critical current under 8 T at 20–65 K with 1.2 MeV proton irradiation at three different fluences; (**a**–**d**) correspond to 20 K, 30 K, 50 K, and 65 K, respectively [46].

As indicated in Figure 4, under c-axis 1.2 MeV proton irradiation at a fluence of 1 × 10^16^ ions/cm^2^, the $J_{c}$ of the REBCO sample increases by approximately 2.6 times at 20 K and 8 T [46]. However, as the temperature rises, the thermal energy acquired by vortices enables them to escape from weak pinning centers, leading to a gradual decline in the enhancement along the c-axis. Therefore, compared to other irradiation methods, defects introduced by proton irradiation are generally more effective at lower temperatures (<30 K). Additionally, due to the relatively high penetration depth of protons during irradiation, defects can be uniformly distributed throughout the material, minimizing the anisotropy typically associated with directional irradiation. The resulting defects are numerous and randomly dispersed, contributing to strong isotropic pinning.

**Figure 5:**
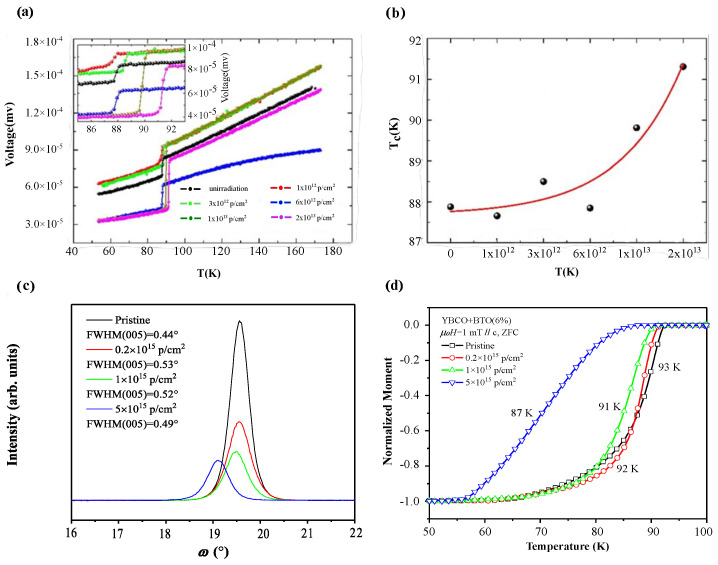
(**a**) Voltage–temperature relationship under different irradiation fluence; (**b**) Dependence of superconducting transition temperature on proton irradiation fluence [40]; (**c**) Rocking curves of samples irradiated with 60 keV protons at various fluence; (**d**) Variation of $T_{c}$ with different 60 keV proton irradiation fluence [49].

The primary defects induced by proton irradiation involve partial transformation from the superconducting orthorhombic phase to the semiconducting tetragonal phase, as well as the formation of oxygen vacancies and interstitial oxygen in the conductive CuO_2_ planes [47]. The superconducting transition temperature $T_{c}$ of REBCO is largely influenced by oxygen disorder and deficiency. A suitable density and size of defects can enable superconducting materials to exhibit excellent performance under high magnetic fields and high current densities. However, high-fluence irradiation may cause severe lattice degradation, including the aggregation of point defects, extension of dislocations, and destruction of the crystal structure. These degradations not only affect the microstructure of the material but may also lead to a reduction in the superconducting transition temperature $T_{c}$. According to the illustration in Figure 5a, under low irradiation fluence, $T_{c}$ exhibits oscillatory behavior; with further increase in fluence, $T_{c}$ gradually increases [40], and Figure 5b presents the fitting curve based on experimental data. Since the displacement energy of oxygen atoms in Cu-O chains is significantly lower than that in the CuO_2_ planes, oxygen defects occurring in the CuO_2_ planes can lead to a decrease in $T_{c}$ [48]. Therefore, at low fluence, oxygen defects are mainly located in the Cu-O chains. When the fluence increases to $1\times 10^{14}$, $T_{c}$ decreases significantly [47], as lattice damage begins to occur, altering lattice parameters. Although the full width at half maximum (FWHM) increases slightly after irradiation, indicating a limited effect on crystal orientation, the destruction of the lattice results in significant oxygen loss and a substantial decrease in $T_{c}$; Figure 4c,d show where $T_{c}$ decreases by 5 K under excessive fluence.

For REBCO coated conductors, the overall impact of proton irradiation on superconducting performance is closely related to the incident energy. Although the energy required to produce an equivalent level of EEL varies among samples, a qualitative comparison can still be made by dividing typical proton irradiation conditions into low- and high-energy regimes. To provide a clearer view of their respective effects, Table 1 [46,50,51,52] summarizes the characteristic behaviors of low- and high-energy proton irradiation based on empirical observations.

Proton irradiation introduces controllable and relatively homogeneous defects in REBCO coated conductors, with the defect type and density strongly dependent on the proton energy and fluence. As summarized in Table 1, low-energy proton irradiation (typically below 5 MeV) results in localized damage dominated by NEL processes. Such irradiation mainly produces point defects and dislocation loops concentrated near the film surface or the Ag capping layer, leading to mild lattice expansion and local microstrain. Under moderate fluence, $T_{c}$ remains nearly unchanged, while weak point pinning centers slightly enhance $J_{c}$ at low magnetic fields and temperatures. Therefore, low-energy proton irradiation is often applied in surface damage simulations and radiation-hardness testing of coated conductors.

In contrast, high-energy protons (10–200 MeV) penetrate through the entire superconducting layer, where EEL dominates the interaction mechanism. This leads to the formation of nanoscale dislocation clusters and sparse columnar or chain-like defects that act as effective bulk pinning centers. Consequently, $J_{c}$ can be markedly enhanced in the high-field regime, although excessive irradiation may induce severe lattice distortion and oxygen deficiency, resulting in a significant decrease in $T_{c}$. Overall, proton irradiation offers a high degree of controllability and reproducibility, providing a practical approach to optimizing flux pinning while maintaining structural integrity within an appropriate fluence window.

## 4. Fast Heavy Ion Irradiation

### 4.1. Defect Characteristics of Heavy Ion Irradiation

**Figure 6:**
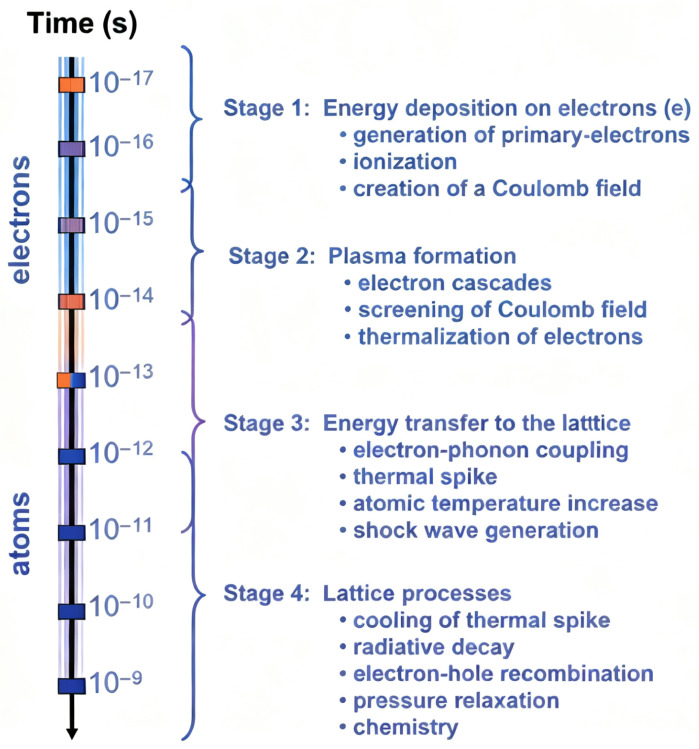
Schematic diagram of latent track formation by swift heavy ion interaction with materials [55].

Due to the extremely high energy of heavy ions, EEL dominates, resulting in a series of unique defect structures in the material that significantly contribute to flux pinning in superconductors [53]. When heavy ions penetrate the material, intense electronic excitations occur along their trajectories, causing instantaneous local melting and resolidification in the track region. This process forms columnar amorphous or damaged zones with diameters of several nanometers and extended depths. Permanent structural damage known as latent tracks can form along the ion incidence path. The formation of latent tracks can be quantitatively described by the thermal-spike model [54]. This process involves two steps: first, the ion transfers energy to the target atoms’ electrons along its path, causing extensive electron ionization and excitation; subsequently, electrons transfer energy to lattice atoms through electron-phonon interactions, leading to a rapid, localized temperature rise along the trajectory sufficient to cause melting. The molten region is then rapidly quenched at a rate of approximately 10^13^ K/s, ultimately forming the latent tracks, as schematically illustrated in Figure 6.

**Figure 7:**
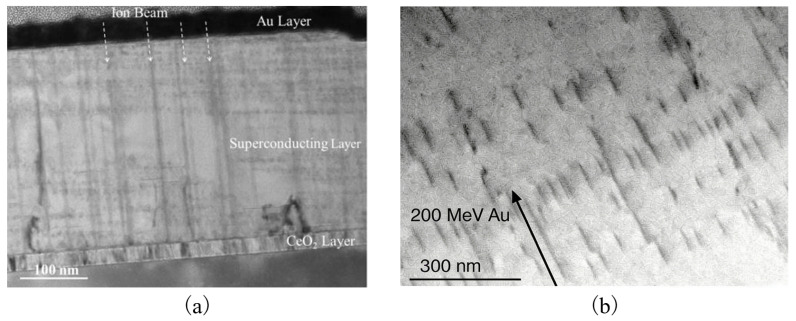
(**a**) TEM image of continuous columnar defects produced by irradiation with Ta ions at a fluence of $1.0\times 10^{11}$ ions/cm^2^ and energy of 1.9 GeV [60]. (**b**) Transmission electron microscopy image showing discontinuous columnar defects in an irradiated MgB_2_ crystal [61].

Nanometer-scale columnar defects and one-dimensional continuous damage tracks are thus formed in the material, manifested as a series of discontinuous or continuous atomic displacements and clusters of point defects over a certain length, with volumes small but lengths reaching several hundred nanometers [56]. One key parameter for defect strength is the volume of vortex lines trapped by the defect. For columnar defects produced by heavy ions, this essentially corresponds to their length. For zero-dimensional defects (such as point pinning centers, interstitial atoms), the pinning potential is approximately $\xi^{3}$ (where ξ is the coherence length), while for higher-dimensional defects, the pinning potential can be expressed as $\xi^{2}L$, where *L* is the trapping length [57]. Due to the small value of ξ, the pinning potential generated by point defects is relatively weak. Under relatively high temperatures of 65–77 K, vortices have sufficient thermal energy to overcome these weak pinning potentials, causing these weak pinning centers to become ineffective [56]. At high temperatures, only defects with sufficiently large volumes (and thus strong pinning forces) can effectively contribute [58]. As the pinning potential of small defects is insufficient to trap flux lines. Therefore, the long columnar defects produce strong pinning effects that effectively restrict flux motion. According to the illustration in Figure 7, continuous columnar defects are formed inside superconducting films after 1.9 MeV Ta ion irradiation. When these continuous columnar defects align with the external magnetic field direction, they serve as very strong flux pinning centers, significantly enhancing the critical current density $J_{c}$ under high magnetic fields. However, since the latent track regions are fully damaged and locally amorphized, the intrinsic current density of the superconducting films decreases, and the critical transition temperature $T_{c}$ also reduces, generally decreasing further with increasing irradiation fluence [59].

The size of columnar defects formed by heavy ion irradiation largely depends on the electronic energy loss $S_{e}$. When $S_{e}$ exceeds a certain threshold, continuous columnar defects form within the film, whereas when $S_{e}$ is below this threshold, thinner and spaced discontinuous columnar defects are produced [61,62], as shown in Figure 7b. The formation of discontinuous defects provides strong pinning forces from the columnar defects while reducing the volume of damaged superconducting material, thereby further enhancing the critical current density.

With respect to thermal stability, columnar defects introduced by heavy-ion irradiation generally exhibit high structural robustness. Owing to their formation dominated by intense electronic energy loss, these defects are often associated with locally amorphized or highly disordered regions, rendering them stable over both spatial and energetic scales. Previous studies [63] have shown that under conventional annealing treatments or application-relevant temperature ranges, the positions and overall morphology of columnar defects are largely preserved, although partial structural relaxation or recrystallization within the defect core may occur. As a result, heavy-ion-induced columnar defects are widely regarded as thermally stable pinning centers, particularly suitable for long-term operation under high-temperature and high-field conditions.

### 4.2. Differences Among Various Configurations of Columnar Defects

The strength of flux pinning not only depends on the dimension, size, density, and distribution of defects but also significantly on their orientation. Three-dimensional pinning centers such as nanoparticles and zero-dimensional pinning centers like point defects exhibit no preferred orientation in flux pinning, resulting in isotropic pinning forces with respect to magnetic fields from all directions. In contrast, one-dimensional columnar defects produced by heavy ion irradiation generate strong flux pinning when the magnetic field aligns with their longitudinal axis; thus, the orientation of these one-dimensional artificial pinning centers determines the optimal direction for flux pinning [45,64]. Therefore, by adjusting the direction of ion irradiation, the critical current density under different magnetic field angles can be tuned to meet various application requirements.

**Figure 8:**
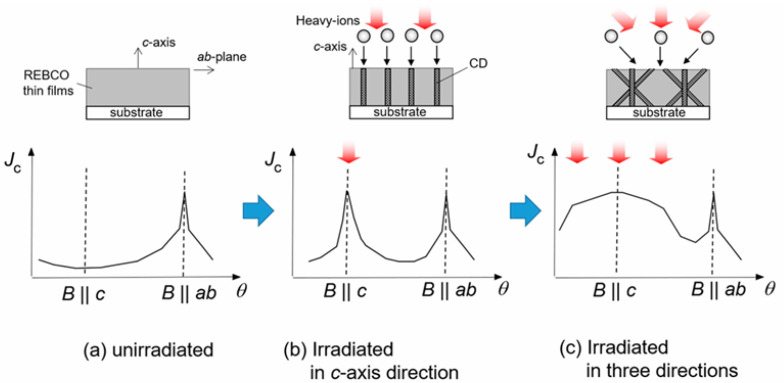
Schematic diagram illustrating the modification of $J_{c}$ anisotropy by different irradiation directions [65].

Subsequently, researchers began performing heavy ion irradiation on superconducting films at various angles, aiming to enhance $J_{c}$ values at different angles by generating crossed columnar defects with different configurations [65], as schematically illustrated in Figure 8. Tetsuro Sueyoshi et al. conducted crossed irradiation on YBCO along the *c*-axis at ±10° and found that at relatively low magnetic fields, an additional peak near the *c*-axis appeared, with the $J_{c}\left(\theta \right)$ peak height exceeding that of samples with parallel columnar defects [66].

**Figure 9:**
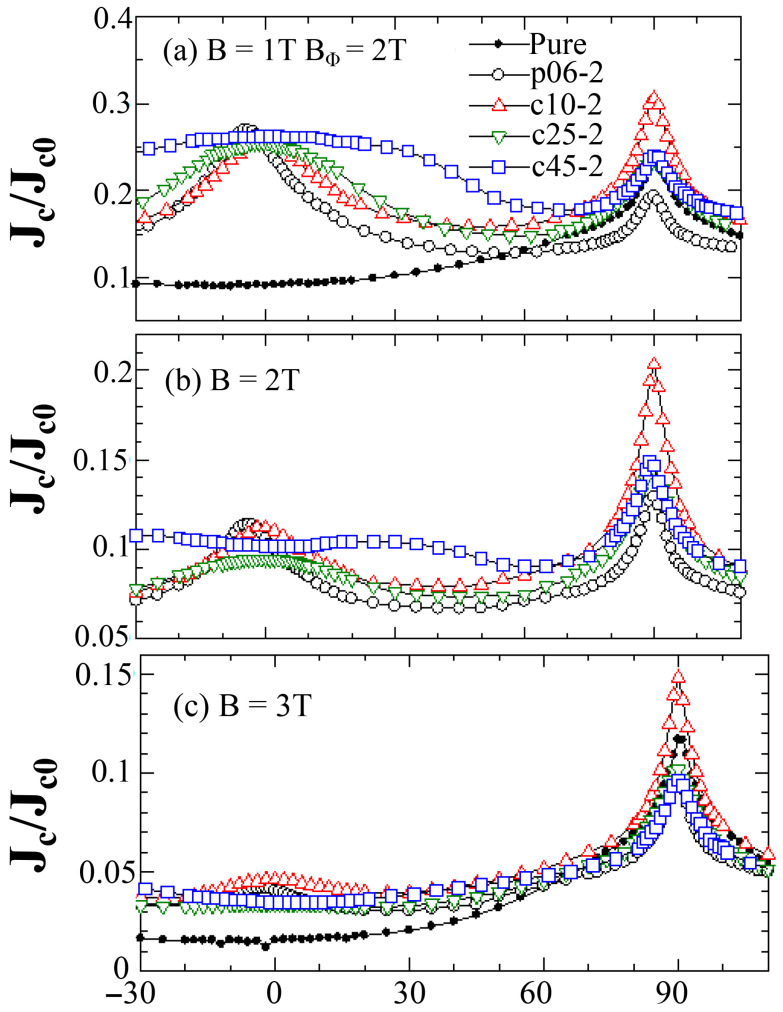
Normalized critical current density $J_{c}$ versus magnetic field angle dependence under different crossed columnar defect configurations at various magnetic fields (c10-2: $\theta_{i}=\pm 10^{\circ }$, c25-2: $\theta_{i}=\pm 25^{\circ }$, c45-2: $\theta_{i}=\pm 45^{\circ }$, p06-2: parallel CD configuration with $\theta_{i}=6^{\circ }$, and Pure: unirradiated samples) [70].

The effects of columnar defects at different angles in a double-peak configuration vary significantly. The crossing angle markedly influences the shape of the additional peak near B ‖ c; larger crossing angles broaden the $J_{c}$ peak width, improving sample performance over a wider magnetic field angular range. The normalized $J_{c}$ versus angle curves (Figure 9) display a single peak near the *c*-direction when the crossing angle $\theta_{i}\leq \pm 25^{\circ }$, whereas a double peak emerges at $\theta_{i}=\pm 45^{\circ }$. At lower magnetic fields, $J_{c}$ is also enhanced in the angular region from $-45^{\circ }$ to $45^{\circ }$, but decreases rapidly with increasing field. The B ‖ c condition causes double peaks near irradiation angles of ±45°. This crossing phenomenon is attributed to differences in vortex pinning states between B ‖ c and the irradiation angles; the ±45° crossed columnar defects fail to provide correlated vortex pinning along the *c*-axis, causing the loss of correlated pinning along the long axis at B ‖ c.

Tetsuro Sueyoshi et al. also investigated a three-peak configuration in YBCO samples irradiated at 0° and ±45° relative to the *c*-axis, finding that the critical current density $J_{c}$ for the three-peak configuration was higher than that of any other configuration across all magnetic fields, and the *n*-value of the three-peak configuration was significantly enhanced [67].

**Figure 10:**
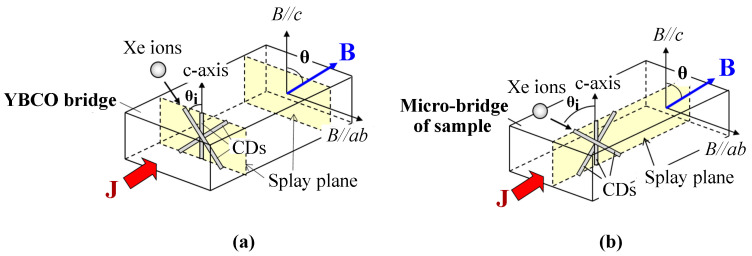
Schematic diagrams of two types of three-peak configurations: (**a**) standard three-peak configuration [65], (**b**) another three-peak configuration [71].

**Figure 11:**
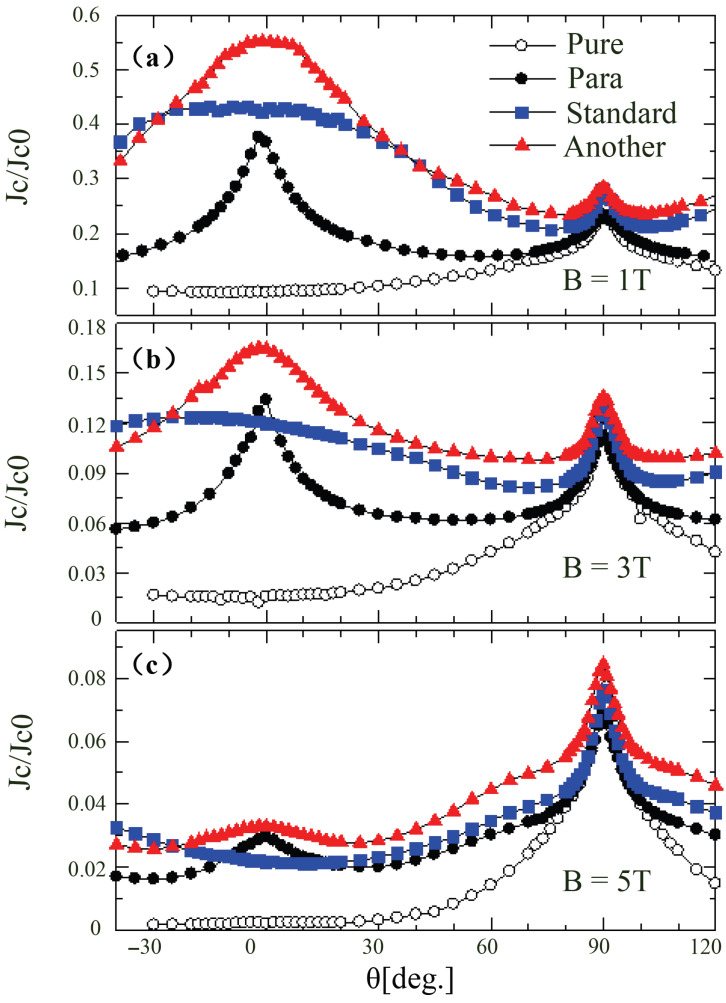
Dependence of normalized critical current density $J_{c}$ on magnetic field angle under various configurations [72] (Pure: unirradiated samples, Para: parallel CD configuration with $\theta_{i}=0^{\circ }$, Standard: standard trimodal configuration, and Another: another trimodal configuration).

Due to the different planar orientations formed by the columnar defects, two types of three-peak configurations are identified in Figure 10, and these two configurations have distinct impacts on superconducting performance. The critical-current density variations of two three-peak configurations after irradiation as a function of magnetic-field angle are shown in Figure 11, which demonstrates the performance differences between the two configurations under different magnetic fields. For the alternative configuration, the standard configuration forms a plateau-like curve in the B  ‖  c direction, while the other configuration produces a peak with higher performance than the standard one. In the standard three-peak configuration, when B  ‖  c, the crossed planes formed by the columnar defects are parallel to the Lorentz force, causing vortices to potentially slide along the tilted flux lines, thereby reducing pinning efficiency [68]. In contrast, the pinning planes formed by the other three-peak configuration are perpendicular to the Lorentz force direction, allowing vortices to cross the intersecting flux lines under the Lorentz force, thus providing stronger vortex pinning in the plane parallel to the transport current direction when B  ‖  c. Furthermore, even when the magnetic field tilts away from the *c*-axis, the $J_{c}$ of the alternative three-peak configuration remains higher than that of the standard configuration, possibly due to vortex entanglement formed within the grid of planes tilted relative to the magnetic field, which suppresses vortex motion in this grid structure [69].

### 4.3. Effects of Heavy Ion Irradiation on Performance

At low temperatures, the thermal activation energy is low, the superconducting condensation energy is high, and thermal fluctuations are weak, enabling some weak pinning centers (such as intrinsic point defects, interface roughness, small-scale stress regions, etc.) to exert strong pinning effects and effectively pin vortices. Therefore, the introduction of additional columnar defects provides limited enhancement at low temperatures. Moreover, vortex lines exhibit strong rigidity at low temperatures, reducing the probability of escaping around defects but also diminishing defect selectivity, leading to smaller improvements under these conditions [73,74].

**Figure 12:**
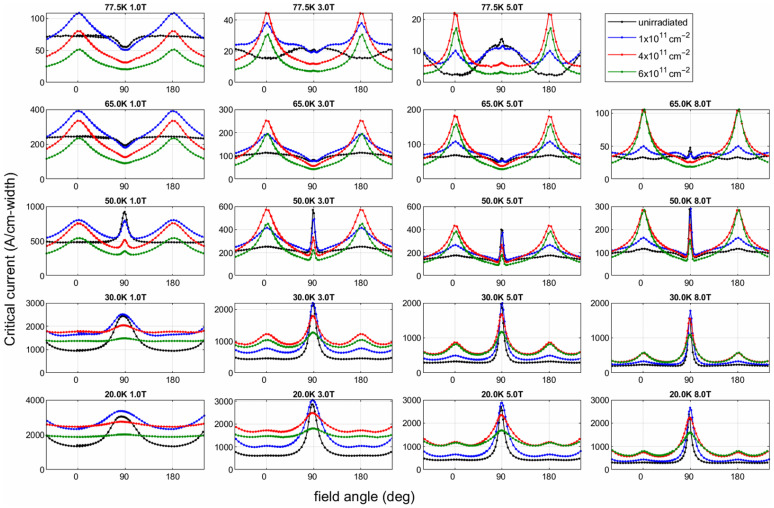
Angular dependence of critical current for samples irradiated along the *c*-axis with 100 MeV Ag ions at different fluence, measured under external magnetic fields of 1 T, 3 T, 5 T, and 8 T over the temperature range of 20 K to 77 K [75].

As shown in Figure 12, Nicholas M. Strickland et al. [75] investigated superconducting thin films irradiated with 100 MeV Ag ions and measured the angular dependence of the critical current under magnetic fields of 1, 3, 5, and 8 T over a temperature range from 20 K to 77 K. Since the irradiation was performed perpendicular to the film surface, the analysis mainly focuses on the enhancement of the critical current at 0°, corresponding to the B ‖ c configuration. The results indicate that at 20 K, a pronounced enhancement of the critical current is observed for all irradiation fluences in the perpendicular field orientation, with the enhancement approaching a factor of two as the magnetic field increases. When the temperature is raised to 50 K, samples subjected to high irradiation fluence may exhibit inferior performance compared with the pristine sample at low magnetic fields; however, with increasing field strength, columnar defects become dominant, and the high-fluence irradiated samples show nearly a twofold enhancement at 8 T. Upon further increasing the temperature to 65 K, the enhancement at high fields exceeds a factor of two. In contrast, at low magnetic fields below the matching field, vortices cannot fully occupy all available defects, resulting in reduced pinning efficiency. Under strong magnetic fields of 5 and 8 T, these defects are effectively utilized, giving rise to a pronounced enhancement of the critical current.

However, since heavy ion irradiation was performed only along the *c*-axis, the anisotropy of the columnar defects causes their pinning contribution in the ab-plane to continuously decrease with increasing temperature. Moreover, compared to the unirradiated samples, the superconducting volume is reduced, resulting in a significant decrease in the intrinsic pinning and the critical current density $J_{c}$ along the ab-direction at high temperatures relative to the original unirradiated samples.

For low-energy heavy ions dominated by nuclear energy loss, excessive localized damage may instead deteriorate superconducting properties. Pęczkowski et al. reported that 250 keV Ne^+^ irradiation on 2G HTS tapes induced severe degradation of $I_{c}$ and $T_{c}$ [76]. The low-energy Ne ions primarily generated dense displacement cascades and point defects confined to the near-surface region, which disrupted the superconducting continuity without forming effective pinning centers. This result highlights that nuclear-energy-loss-dominated low-energy heavy-ion irradiation tends to cause performance deterioration rather than flux pinning enhancement, in sharp contrast to high-energy heavy-ion irradiation that produces extended columnar defects.

It should be noted that heavy-ion irradiation produces distinctly different defect types and pinning characteristics depending on the energy range, with their morphology and density strongly dependent on the ion energy and irradiation fluence. To better understand the effects induced by irradiations at different energies, these impacts have been summarized in Table 2 [77,78,79,80]. Low-energy heavy ions predominantly induce displacement damage and amorphous regions near the surface, dominated by NEL, whereas high-energy heavy ions primarily cause EEL, forming columnar defects that penetrate the full thickness of the superconducting layer. These differences determine their distinct influences on flux pinning strength, anisotropy, and the field- and temperature-dependence of the critical current density $J_{c}$.

## 5. Neutron Irradiation

### 5.1. Types and Characteristics of Defects Induced by Neutron Irradiation

Neutron irradiation, as an expensive irradiation method, is difficult to apply to long-length tapes and further commercialization. However, it can produce randomly distributed collision cascade defects without preferential orientation (high defect concentration microscale regions formed by MeV-level neutron collisions generating high-energy atomic recoils), and at relatively low fluences, the superconductivity of samples remains almost unchanged [81]. Additionally, REBCO, as an ideal superconductor for compact fusion reactor magnetic confinement systems [82], inevitably encounters high-energy neutron flux during fusion device operation. These neutrons induce microstructural damage inside the REBCO superconductor. Therefore, although neutron irradiation cannot be widely applied, it serves as an important tool for studying flux pinning.

Neutrons are neutral particles, unaffected by Coulomb screening, and have strong penetration ability, allowing them to penetrate deep into materials. They mainly introduce defects through elastic and inelastic collisions [26,83]. Elastic scattering is the most common mechanism when neutrons interact with material atoms. During elastic collisions, atoms are displaced from their equilibrium positions, forming point defects. The impacted atom becomes a primary knock-on atom (PKA), which further induces collision cascades, producing a series of vacancies, interstitials, dislocations, and other defects [84]. Defects caused by elastic scattering are generally dispersed and numerous, with a strongly random distribution [85]. Although these defects affect material properties, under low-fluence irradiation conditions, the defects from elastic scattering are few and typically cause only minor perturbations to the material structure. However, under high-fluence irradiation, the accumulation of elastic scattering defects may significantly degrade superconducting performance.

Inelastic scattering involves energy exchange between neutrons and material atoms, producing small cluster defects that are often more complex than point defects generated by elastic scattering. Neutron energy transfer causes larger atomic displacements, resulting in defect clusters composed of multiple vacancies and interstitials. These clusters cause considerable disturbance to the lattice structure and significantly impact the microstructure of the superconductor. Neutrons undergo multiple scattering events and induce various defect types through both elastic and inelastic processes, typically including point defects, small cluster defects, and atomic displacements.

Although the defect structures introduced by neutron irradiation are not clearly defined, only the density of the largest defects (collision cascade defects) is well known [86], while numerous smaller defects with unknown densities are also generated [87]. It is clear that all defects are randomly distributed and uncorrelated, with no preferential orientation [88]. Moreover, neutron irradiation can introduce randomly distributed spherical pinning centers with diameters on the nanometer scale [89,90], which can enhance sample performance over a broad magnetic field and temperature range. Since the size of neutron-induced defects roughly matches the coherence length of second-generation high-temperature superconducting films, significant improvements in critical current density can be achieved when the initial intrinsic defect concentration is low; however, if the total defect density becomes too high, the critical current density decreases [91].

**Figure 13:**
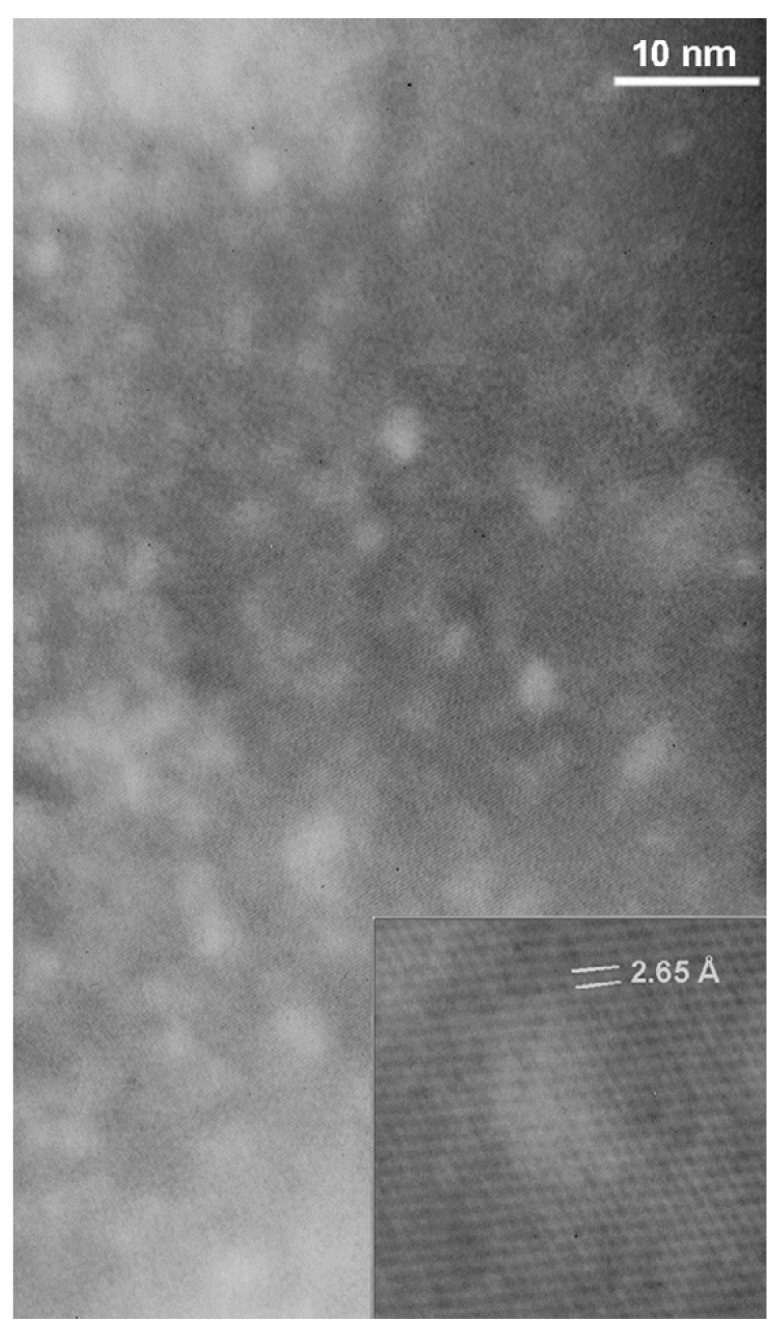
TEM image of a neutron-irradiated MgB_2_ crystal. The inset shows an enlarged view of a defective region, where the lattice fringes lose coherence due to the presence of a localized amorphous area [92].

Transmission electron microscopy provides direct evidence of the defect morphology introduced by neutron irradiation. As shown in the Figure 13 image, neutron-irradiated MgB_2_ exhibits a homogeneous distribution of nanoscale defect regions embedded within an otherwise well-ordered crystalline matrix. These defects appear as light-contrast zones with typical lateral sizes of approximately 3–4 nm, comparable to the superconducting coherence length. High-resolution imaging (inset) reveals a loss of lattice fringe coherence within these regions, while well-defined lattice planes remain visible in the surrounding matrix, indicating the formation of localized amorphous zones rather than extended dislocation networks. Such nanometric amorphous regions are characteristic of displacement cascades generated by neutron-induced elastic collisions and are absent in unirradiated samples. Their density increases systematically with neutron fluence, confirming their irradiation origin. Owing to their size and spatial distribution, these cascade-induced defects are highly effective as flux pinning centers, contributing to the enhancement of the critical current density under high magnetic fields.

The cascade cores themselves tend to be more resistant to complete thermal annihilation, such that the overall spatial distribution of neutron-induced damage is largely retained after moderate annealing treatments [90]. Consequently, while annealing can reduce the density of the most mobile point defects and weaken the strongest irradiation-induced pinning contributions, a substantial fraction of the background pinning associated with cascade damage often persists. This mixed response to thermal treatment highlights the dual nature of neutron irradiation effects, combining elements of both metastable point-defect pinning and more robust defect clusters, and underscores the importance of considering post-irradiation thermal history when evaluating the long-term performance of neutron-irradiated superconductors [87].

### 5.2. Effects of Neutron Irradiation on Current-Carrying Performance

At high neutron irradiation fluence, the defect density increases significantly, which may lead to the emergence of a “collective pinning” mechanism. Collective pinning is a physical phenomenon in which, when a superconductor contains a high density of defects with weak individual pinning forces, flux vortices are no longer pinned individually by single defects, but are instead confined collectively and cooperatively by a group of defects. This mechanism is particularly prominent at high defect densities because the interactions between defects strengthen, making it difficult for flux lines to move freely within the material [93].

Through collective pinning, the material can sustain higher magnetic fields without flux motion, thereby enhancing the critical current density [94]. This mechanism is especially important after high-fluence irradiation because, as defect density increases, the pinning effect of individual defects on flux lines may become limited, whereas collective pinning can establish a stronger pinning network among defects, further improving the flux pinning capability of the material [74].

**Figure 14:**
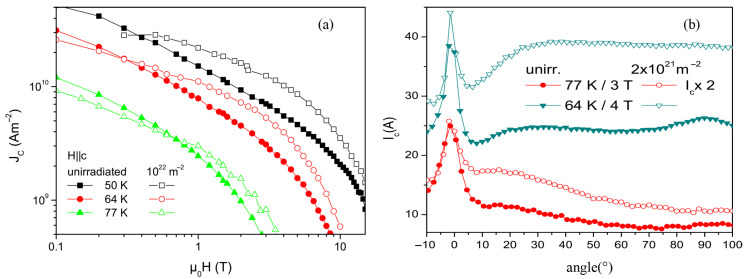
(**a**) Relationship between critical current and magnetic field at different temperatures; (**b**) Relationship between angle and critical current before and after irradiation [91].

The effect of neutron irradiation on $J_{c}$ largely depends on the initial defect structure. When small defects with low density exist prior to irradiation, these defects, though ineffective in enhancing critical current under high temperature and high field conditions alone, can aggregate and grow together with irradiation-induced new defects to form effective pinning centers after neutron irradiation [95]. Figure 14a shows that samples with initially low pinning density exhibit significant enhancement of critical current at high fields after neutron irradiation. Moreover, spherical defects and point defects produced during neutron irradiation are typically distributed randomly within the material. Although these randomly distributed defects may produce varying degrees of flux pinning locally, their overall effect is to enhance the uniform pinning ability of the material. An unirradiated sample in Figure 14b shows a peak at 90°, which disappears after neutron irradiation, and almost no peak exists at 64 K and 4 T. Simultaneously, the performance minimum point (around 9°) significantly increases, and the anisotropy $\gamma_{I_{c}}=I_{c,\max}/I_{c,\min}$ is markedly reduced.

### 5.3. Effects on the Critical Transition Temperature $T_{c}$ and the Irreversibility Line

**Figure 15:**
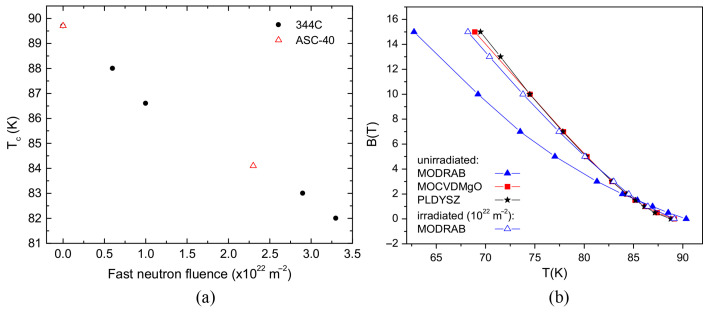
(**a**) Dependence of the transition temperature on the fast neutron irradiation fluence [97]. The samples are two types of coated conductors from American Superconductor Corporation: ASC-40 with a YBCO thickness of 1.2 μm and 344C with a YBCO thickness of 0.8 μm. (**b**) Irreversibility lines of coated conductors fabricated by three different methods after irradiation [91].

Due to the uniformly distributed point defects generated in the superconductor lattice during neutron irradiation, $T_{c}$ exhibits a monotonically decreasing trend with increasing neutron fluence [96]. The anisotropy of the superconducting order parameter is the main reason for the relatively large suppression of $T_{c}$ caused by impurity scattering in cuprates. Since anisotropy of the order parameter is common in superconducting materials, it can be inferred that most superconductors exhibit similar effects on the critical transition temperature $T_{c}$ after neutron irradiation [97]. From the information depicted in Figure 15, the initial transition temperature of both tapes is 89.6 K, and with increasing irradiation fluence, $T_{c}$ of the samples shows an almost linear decrease.

For under-optimized thin film samples, neutron irradiation under appropriate conditions can shift the irreversibility line upward [98]. When the applied magnetic field exceeds a certain threshold between the lower and upper critical fields (H_*c*1_ and H_*c*2_), thermodynamic fluctuations of the superconducting order parameter lead to the depinning of vortices from extended defects. The onset of dissipation is governed by the irreversibility line $H_{\mathrm{irr}}\left(T\right)$, which marks the boundary between the vortex liquid phase and the non-dissipative state, and thus sets the maximum achievable limit [99]. Neutron irradiation significantly increases the defect density in under-optimized samples; therefore, the introduction of these defects into the superconductor causes a notable upward shift of the irreversibility line $H_{\mathrm{irr}}\left(T\right)$ along the temperature axis [45,100].

### 5.4. Energy-Dependent Effects of Neutron Irradiation

Due to their electrically neutral nature, neutrons primarily introduce randomly distributed point defects and small defect clusters through nuclear reactions and displacement cascades. Compared with charged particles, neutron irradiation offers relatively limited flexibility in parameter control, and the resulting defect density is predominantly determined by the irradiation fluence. Consequently, optimization of neutron irradiation parameters focuses on balancing pinning enhancement and superconducting property degradation through careful control of fluence. Moderate neutron fluence can effectively enhance pinning in low critical-current regions and reduce anisotropy, whereas excessive irradiation may cause severe lattice damage and irreversible degradation of superconducting performance.

Neutron irradiation alters the microstructure and superconducting performance of REBCO coated conductors through elastic and inelastic collisions, with the resulting defect morphology strongly dependent on neutron energy. As summarized in Table 3 [101,102,103,104,105], low-energy neutrons (thermal to slow) primarily interact via (n,γ) capture and elastic scattering, introducing mild oxygen vacancies and point defects with minimal lattice distortion. Such low-fluence irradiation has minimal effect on $T_{c}$, and may even enhance $J_{c}$ slightly at low and intermediate magnetic fields due to increased isotropic point pinning.

Conversely, high-energy (fast) neutrons (>0.1 MeV) induce strong atomic displacements and dense collision cascades that lead to a high concentration of cluster defects throughout the superconducting layer. These isotropic defects effectively suppress anisotropy and shift the irreversibility line $H_{\mathrm{irr}}\left(T\right)$ upward, significantly improving high-field $J_{c}$ performance. However, excessive fluence can cause oxygen loss and lattice strain, resulting in a noticeable $T_{c}$ reduction of up to 5–10 K. Overall, neutron irradiation provides a uniform and deeply penetrating damage profile, offering an efficient means to enhance pinning strength and radiation tolerance in REBCO materials.

## 6. Mixed Irradiation

Table 4 summarizes recent research on performance evolution post-irradiation (proton, heavy-ion, neutron), where significant enhancement has been reported. The respective enhancement mechanisms, as elaborated in previous sections, are distinctly tied to the type of irradiation. A key observation is that the substantial performance gain for each ion species is contingent upon a specific temperature or narrow temperature range. Consequently, there exists a notable challenge in achieving wide-ranging, multi-parameter performance improvement through these methods.

Before delving into the details of mixed irradiation, it is essential to compare the characteristics and effects of the individual irradiation techniques that serve as its foundation. Proton, heavy-ion, and neutron irradiation each induce distinct defect landscapes governed by different energy-loss mechanisms. Proton irradiation, dominated by NEL, primarily generates nanoscale point defects that enhance isotropic pinning at low temperatures. In contrast, heavy-ion irradiation, which is governed by EEL, produces extended columnar tracks that provide strong correlated pinning under high magnetic fields but at the expense of increased anisotropy and $T_{c}$ degradation. Neutron irradiation, on the other hand, introduces random cascade defects with deep penetration, leading to moderate improvements in $J_{c}$ and reduced angular dependence.

Table 5 summarizes the comparative features, advantages, and limitations of these irradiation methods, providing a concise framework for understanding how different defect structures contribute to flux-pinning performance. This comparison also highlights why mixed irradiation, combining multiple energy-loss processes, has emerged as a promising approach to constructing multiscale pinning landscapes that balance pinning strength, isotropy, and stability.

Following the comparative analysis summarized in Table 5, it becomes clear that no single irradiation method can simultaneously achieve strong pinning, high isotropy, and minimal degradation of superconducting properties. This realization has motivated the development of *mixed irradiation* approaches, in which two or more particle species are applied either sequentially or concurrently to combine the benefits of different defect types.

Rather than producing a uniform defect population, mixed irradiation aims to construct a controlled hierarchy of defects—ranging from point-like to columnar—each contributing to flux pinning under different magnetic-field and temperature conditions. The essence of this strategy lies in exploiting the complementary energy-loss mechanisms of distinct projectiles: while heavy ions generate correlated columnar tracks through EEL, protons and neutrons primarily introduce dense point or cluster defects via nuclear collisions. The interplay between these mechanisms creates a multiscale defect landscape that can be tuned to specific performance requirements.

In mixed irradiation strategies, particle-specific parametric optimization is further reflected in the deliberate selection of irradiation sequence and synergistic defect design. It is generally considered favorable to first introduce columnar defects via heavy-ion irradiation as a pinning backbone, followed by proton or neutron irradiation to supplement random point defects. Such a sequential irradiation strategy enables complementary pinning at different length scales while mitigating competition between different defect populations, thereby providing an effective pathway toward constructing multiscale pinning landscapes with a certain degree of predictability.

In recent years, numerous studies have confirmed that properly engineered mixed irradiation can yield a more uniform $J_{c}\left(B,\theta \right)$ response and improved flux-creep stability compared with any single-particle irradiation. These findings indicate that mixed irradiation is not merely a combination of effects, but a deliberate design methodology for tailoring the vortex-pinning environment in REBCO conductors.

### 6.1. Dual-Region Enhancement of Critical Current Density

One of the most striking features of mixed irradiation is the so-called *dual-region enhancement* of the critical current density ($J_{c}$). This behavior—characterized by simultaneous improvements in both the low-field and high-field regimes—originates from the complementary nature of the defects produced by different particles. Columnar defects introduced by heavy ions provide strong correlated pinning that dominates under high magnetic fields, whereas the point and cluster defects created by protons serve as numerous weak pinning centers that reinforce the flux-pinning network at lower fields. When these two types of defects coexist, they effectively “fill the gaps” in the pinning landscape, leading to a more uniform and robust flux immobilization.

**Figure 16:**
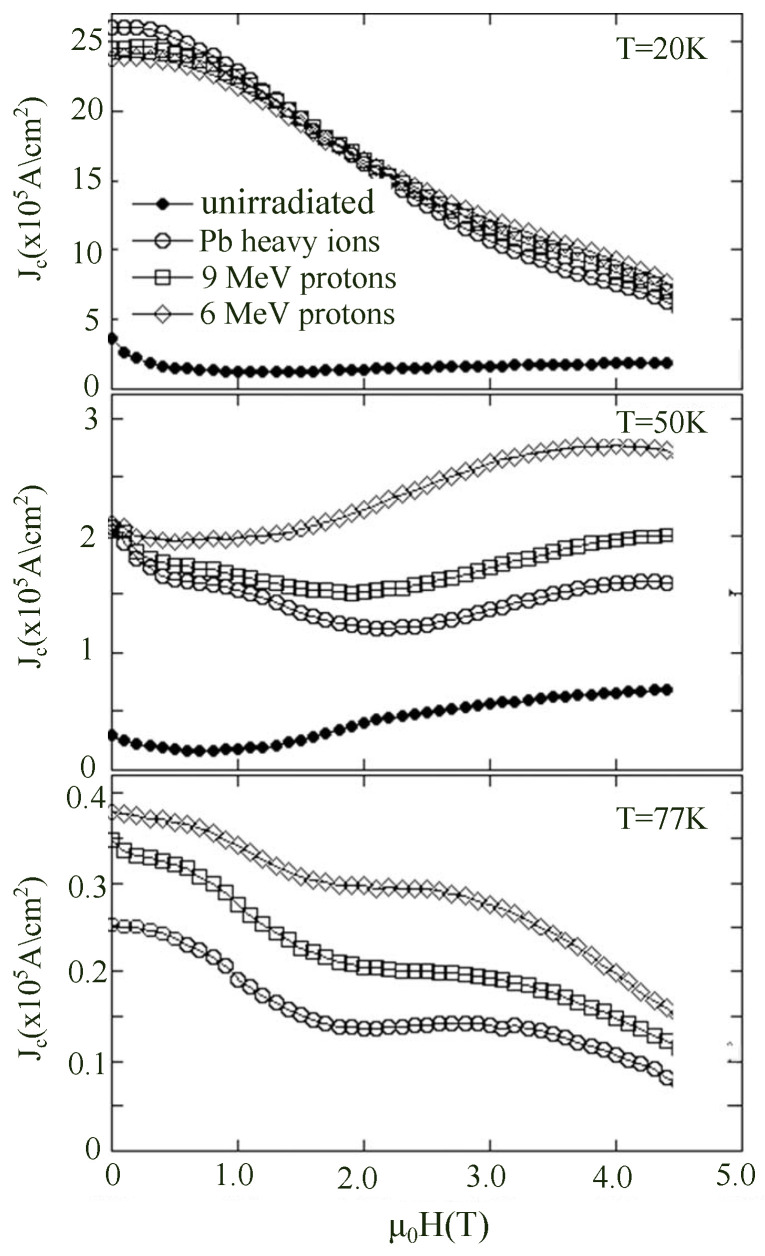
Variation of the critical current density $J_{c}$ with magnetic field applied along the c-axis at T = 20, 50, and 77 K, measured before irradiation, after heavy-ion irradiation, and after sequential heavy-ion and proton irradiations with different proton energies [111].

Experimental work by Hua et al. [111] vividly demonstrated this synergy in YBCO single crystals subjected to sequential heavy-ion and proton irradiation, as schematically illustrated in Figure 16. The resulting $J_{c}\left(H\right)$ curves exhibit enhancements over the entire field range, with particularly strong gains in the high-field region. Interestingly, the improvement does not simply scale with defect density—rather, it reflects an optimized defect hierarchy in which weak point pinning stabilizes vortex lines between the stronger columnar pins. Such cooperative pinning is a recurring theme in modern REBCO optimization.

### 6.2. Significant Reduction of Anisotropy

Mixed irradiation can simultaneously enhance the critical current density ($J_{c}$) and significantly reduce the magnetic-field angular anisotropy of REBCO coated conductors. Due to the different types of defects introduced by protons and heavy ions, their directional selectivity for flux pinning also differs: proton irradiation primarily generates a dispersed distribution of point defects and small dislocation loops, providing nearly isotropic weak pinning, whereas heavy-ion irradiation (e.g., Ag) produces columnar defects oriented along the *c*-axis, exhibiting strong selectivity for perpendicular magnetic fields. Sequential or combined irradiation of the two types can create complementary pinning potentials along different directions, thereby achieving a significant reduction of overall anisotropy.

**Figure 17:**
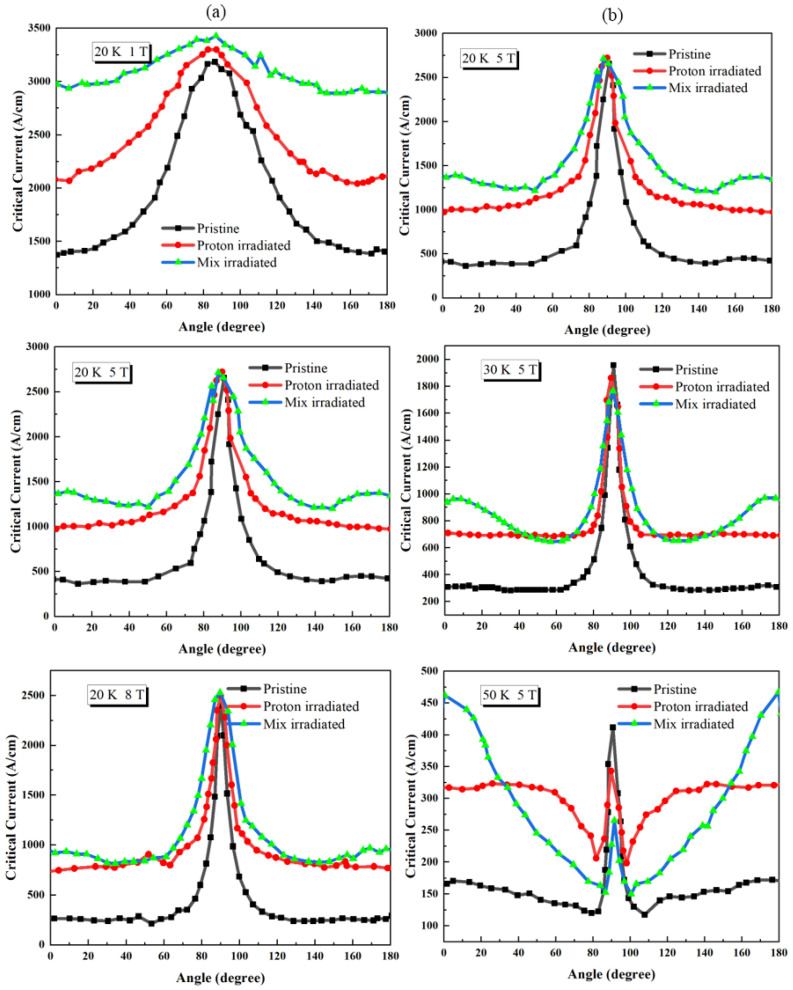
(**a**) Angular dependence of the critical current $I_{C}$ for the pristine and mix irradiated samples at T = 20 K under different magnetic fields (2 T, 5 T, 8 T) (**b**) Angular dependence of the critical current $I_{C}$ for the pristine and mix irradiated samples at B = 5 T under different temperatures (20 K, 30 K, 50 K) [112].

Soman et al. [112] investigated the behavior of REBCO coated conductors sequentially irradiated with 1.2 MeV protons and 125 MeV Ag ions. As shown in Figure 17, the angular distribution of the critical current ($I_{c}$) in the mixed-irradiated samples exhibits a significantly flattened profile in the temperature range of 20–50 K. Compared with samples subjected to single-ion irradiation, the peak associated with the ab-plane is markedly broadened, while the *c*-axis peak remains enhanced. Moreover, at low temperatures, the angular variation of $I_{c}$ is substantially suppressed, indicating a more uniform flux pinning landscape.

With increasing magnetic field, the angular dependence of $I_{c}$ is further weakened, approaching a nearly isotropic distribution at fields above 5 T. This behavior reflects a cooperative pinning mechanism arising from the coexistence of point defects and columnar defects: point defects effectively fill the potential weak-pinning regions between columnar tracks and simultaneously suppress transverse thermal fluctuations of vortices, leading to a more balanced overall pinning potential. This study clearly demonstrates that an appropriate coexistence of point defects and columnar defects can significantly reduce the angular anisotropy of $I_{c}$ in REBCO superconductors and enhance their current-carrying capability under multi-directional high-field conditions. These findings provide important guidance for the stable operation of superconducting tapes in high-field coils, rotating machinery, and magnet applications.

To further clarify the microscopic origin of the broadened effective temperature–field pinning window and the reduced angular anisotropy observed in mixed-irradiated REBCO coated conductors, the cooperative pinning mechanism arising from heterogeneous defects is discussed below. From a microscopic perspective, the expansion of the effective temperature–field pinning window and the reduction of anisotropy in mixed-irradiated REBCO conductors originate from the cooperative interaction among heterogeneous defects with different dimensionalities and characteristic length scales. Point defects introduced by proton irradiation provide isotropic, short-range pinning that is particularly effective at low temperatures, whereas columnar defects generated by heavy-ion irradiation offer strong correlated pinning under high magnetic fields but exhibit pronounced angular selectivity. Randomly distributed cascade defects induced by neutron irradiation further suppress vortex mobility by filling the pinning “gaps” between correlated tracks. When these defects coexist, vortices experience a hierarchical pinning landscape in which different pinning mechanisms dominate in complementary temperature and field regimes. At the atomic and nanometer scale, such heterogeneous defects collectively modulate vortex stiffness and vortex segmentation, leading to enhanced vortex immobilization over a broader temperature–field range and a marked suppression of angular anisotropy.

### 6.3. Defect Competition and Creep Effects

In mixed irradiation systems, the introduction of multiple types of defects not only enhances flux pinning but also induces complex flux dynamics. Different energetic particles produce distinct damage mechanisms in REBCO materials: heavy ions primarily form columnar defects oriented along the *c*-axis through NEL, whereas light particles such as protons, oxygen, or helium mainly introduce randomly distributed point defects and dislocation loops. When these defects coexist, the spatial distribution of the flux-pinning potential transforms from a single well to a composite multi-peak structure, resulting in cooperative or competitive interactions between pinning centers and flux-line motion.

Haberkorn et al. [113] systematically investigated the critical current and creep properties under a composite pinning landscape in GdBa_2_Cu_3_O_7−*δ*_ coated conductors using 6 MeV oxygen-ion mixed irradiation. They found that after oxygen-ion irradiation on tapes containing intrinsic nanodefects, the $J_{c}$ was significantly enhanced in the low-field region, while the flux creep rate S(T) decreased at low temperatures but increased again at higher temperatures, exhibiting a characteristic “dual-region behavior.” This observation indicates that although point defects can compensate for the weak regions between columnar pinning sites, an excessive density of point defects may weaken the correlated pinning effect of columnar defects, thereby increasing the probability of thermally activated flux-line hopping.

Similar competitive behavior also occurs in sequential irradiation systems. Keppert et al. [114] observed in He-ion-irradiated YBCO thin films that point defects introduced during the early irradiation stage can temporarily enhance pinning strength, but during subsequent annealing or aging, some defects migrate or agglomerate, leading to a decrease in $J_{c}$ and a recovery of the flux creep rate. This indicates that point defects possess high mobility and dynamic evolution characteristics, whereas columnar defects are relatively stable. The aging competition between these two defect types directly affects the long-term stability of mixed-irradiated samples.

From the perspective of energy-barrier distribution, the competition between different defect types can result in a multi-scale distribution of creep barriers. A moderate density of point defects can broaden the barrier distribution and reduce the correlation between flux lines, promoting enhanced pinning at low temperatures. However, when the density of point defects is excessive, the system enters a weak-pinning regime, leading to flux decoupling and increased creep rates at high temperatures.

The review by Nicholls et al. [115] also pointed out that this behavior is closely related to the thermal stability of defects formed via different energy-loss mechanisms: point defects dominated by EEL are more prone to migration and recovery, whereas columnar tracks produced by NEL can remain stable over extended periods. Therefore, the optimization of mixed irradiation should strike a balance between “enhancing low-temperature pinning” and “maintaining high-temperature stability,” achieving synergy between the two types of defects through appropriate fluence and energy allocation, rather than allowing competition.

## 7. Conclusions and Future Perspectives

With the increasing demand for superconducting coated conductors, particularly REBCO coated conductors, enhancing their superconducting performance under extreme conditions such as high magnetic fields and low temperatures has become a critical challenge. Mixed irradiation, as an emerging approach, can leverage the advantages of different irradiating particles to introduce multiple types of defects with controllable scales, enabling precise modulation of the flux-pinning landscape and offering significant potential for performance improvement.

Nevertheless, although mixed irradiation shows promising prospects in enhancing the overall pinning properties of REBCO superconducting films and coated conductors, research in this area remains exploratory and faces numerous scientific and engineering challenges. Firstly, the synergistic rules governing different particle types and energy-loss mechanisms are not yet fully understood. Protons, neutrons, and fast heavy ions deposit energy through distinct pathways in the material, and variations in the ratio of electronic to NEL lead to strongly nonlinear relationships in defect formation mechanisms and morphological distributions. Currently, systematic experimental and theoretical studies on the interactions, reconstruction, and stability of defects under mixed irradiation are still lacking.

Secondly, the process controllability and reproducibility of mixed irradiation remain critical issues limiting its practical application. Variations in particle flux, energy, and irradiation sequence can result in markedly different defect evolution pathways. For example, applying high-energy heavy-ion irradiation followed by light proton irradiation may cause point defects around columnar defects to redistribute or agglomerate, altering the original pinning potential landscape. Therefore, establishing tunable irradiation models and a predictive framework for defect evolution under mixed irradiation represents a pressing scientific challenge.

At the same time, potential side effects and cumulative damage to superconducting properties must be carefully considered. While mixed irradiation enhances flux pinning, it may also induce undesirable effects such as disruption of current-carrying channels, reduction of the superconducting transition temperature, or degradation of interfacial layers. Achieving an optimal balance among defect density, type, and size is a key direction for future materials design and process optimization.

Looking forward, research on mixed irradiation is expected to shift from an “empirically driven” approach to a “mechanism-guided, defect-oriented design” paradigm. On one hand, advanced characterization techniques such as in-situ transmission electron microscopy, three-dimensional atom probe tomography, and X-ray coherent diffraction imaging can enable real-time or three-dimensional observation of defect evolution under different irradiation sequences. Importantly, the integration of these structural probes with local superconducting characterization techniques, such as magnetic force microscopy (MFM), scanning Hall probe microscopy, and scanning SQUID microscopy, remains largely unexplored for irradiated REBCO films. Such local probes are uniquely suited to directly visualize vortex distributions and local pinning strength, and could provide missing quantitative links between three-dimensional defect morphology and spatially resolved superconducting responses.

On the other hand, combining multi-scale simulations with machine learning methods holds the potential to establish quantitative correlations between particle energy-loss distributions, defect morphologies, and critical current responses, thereby enabling more precise engineering of the pinning landscape. Furthermore, integrating mixed irradiation strategies with nano-doping, interface engineering, and chemical strain modulation could create synergistic systems with multi-dimensional pinning centers, further enhancing the performance of REBCO materials under high magnetic fields, elevated temperatures, and complex operating conditions.

Overall, mixed irradiation not only provides a novel approach for enhancing superconducting performance but also serves as an important platform for exploring the interplay between irradiation-induced defects and energy-loss mechanisms. With the continuous improvement of experimental precision and theoretical modeling capabilities, this field is expected to develop a systematic research framework that can guide the defect-oriented design of next-generation high-performance superconducting tapes and devices.

## Figures and Tables

**Table 1:** Comparison between low-energy and high-energy proton irradiation on REBCO coated conductors.

Comparison Item	Low-Energy Proton Irradiation	High-Energy Proton Irradiation
Typical energy range	0.1–5 MeV	10–200 MeV
Dominant energy loss mechanism	Nuclear energy losses are absolutely dominant	Electronic energy loss dominates; nuclear loss contributes slightly but produces discrete severe damage
Main defect types	Mainly point defects (vacancies, interstitials), possibly aggregating into dislocation loops	Nanoscale dislocation clusters, sparsely distributed columnar or chain-like defects
Defect distribution characteristics	Localized shallow distribution, with damage concentrated on the film surface or near the protective layer	Irradiation penetrates the entire superconducting layer (when the film is thin), and the defect distribution is relatively uniform
Effect on lattice structure	Local lattice expansion and microstrain increase; lattice parameter slightly enlarged	The lattice parameters and microstrain increase more with low-energy protons, but the overall lattice strain remains small
$T_{c}$ variation trend	Remains unchanged at lower fluence, then decreases linearly	$T_{c}$ As the fluence increases, it generally shows a linear decreasing trend
$J_{c}$ variation trend	$J_{c}$ remains nearly constant at low fluence; weak point pinning centers slightly enhance $J_{c}$ at low fields; improvement limited at high fields	Strong bulk pinning centers generated; $J_{c}$ significantly enhanced under high fields and low temperatures, but decreases at excessive fluence
Flux pinning characteristics	Dominated by zero-dimensional pinning (point defects)	Enhanced one-dimensional or quasi-one-dimensional pinning (defect chains), improving high-field performance
Main application target	Surface damage simulation and low-energy particle shielding studies	High-field performance optimization and irradiation tolerance evaluation
Typical experimental result	$J_{c}$ enhancement limited under low-field and low-temperature conditions	$J_{c}\left(B\right)$ significantly enhanced in high-field region with stronger pinning force

**Table 2:** Comparison between low-energy and high-energy heavy-ion irradiation on REBCO coated conductors.

Comparison Item	Low-Energy Heavy-Ion Irradiation	High-Energy Heavy-Ion Irradiation
Typical energy range	100 keV–5 MeV	100 MeV–1 GeV
Dominant energy loss mechanism	Dominated by NEL near surface; electronic loss plays a secondary role	Strongly dominated by EEL, generating high ionization and displacement cascades
Main defect types	Surface amorphous layer, point and cluster defects	Columnar defects and amorphous tracks, sometimes discontinuous or splayed
Defect distribution characteristics	Localized in near-surface region; shallow penetration (tens of nm)	Penetrates through entire superconducting layer; high uniformity of columnar tracks
Effect on lattice structure	Surface amorphization and increased disorder; partial oxygen depletion	Formation of extended amorphous columns (5–10 nm dia., 100–500 nm long); lattice distortion but overall crystalline framework retained
$T_{c}$ variation trend	Slight decrease due to local disorder and oxygen loss	Slight to moderate $T_{c}$ reduction (1–5 K) depending on fluence
$J_{c}$ variation trend	Slight $J_{c}$ enhancement at low field due to increased point pinning	Significant $J_{c}$ enhancement (2–3×) under high magnetic fields (>5 T) and at low *T*
Flux pinning characteristics	Dominated by point and short-range pinning; isotropic pinning behavior	Strong correlated pinning along *c*-axis; reduced anisotropy; enhanced high-field pinning force
Main application target	Surface modification and defect-engineering studies	High-field magnet design; directional correlated pinning optimization
Typical experimental result	Partial amorphization at surface; limited $J_{c}$ improvement	Formation of uniform columnar tracks observed by TEM; strong $J_{c}$ enhancement verified experimentally

**Table 3:** Comparison between low-energy and high-energy neutron irradiation on REBCO coated conductors.

Comparison Item	Low-Energy Neutron Irradiation	High-Energy Neutron Irradiation
Typical energy range	Thermal–slow neutrons (<0.5 eV)	Fast–high-energy neutrons (>0.1 MeV)
Dominant energy loss mechanism	Mainly neutron capture (n,γ) reactions and elastic scattering	Inelastic scattering and knock-on collisions causing atomic displacements
Main defect types	Oxygen vacancies, point defects, and light cluster formation	Dense displacement cascades, defect clusters, and nanometric collision cascades
Defect distribution characteristics	Relatively uniform but low-density damage; localized near oxygen sublattice	Uniform volumetric damage through entire thickness; deeper penetration and higher defect density
Effect on lattice structure	Minor oxygen deficiency and local lattice expansion; limited strain	Strong atomic displacements causing microstrain and lattice distortion at high fluence
$T_{c}$ variation trend	Nearly unchanged at low fluence; slight reduction with increasing fluence	Noticeable decrease in $T_{c}$ (up to 5–10 K) at high fluence due to oxygen loss and disorder
$J_{c}$ variation trend	Slight enhancement at low and intermediate fields due to increased point pinning	Significant enhancement of $J_{c}$ under high magnetic fields; saturation or decline at excessive fluence
Flux pinning characteristics	Random isotropic pinning centers formed by point defects	Enhanced isotropic pinning and suppression of anisotropy; improved vortex stability
Main application target	Simulation of reactor neutron environments; stability evaluation	Enhancement of high-field performance and radiation-hard design for magnets
Typical experimental result	Moderate $J_{c}$ enhancement and nearly constant $T_{c}$ at low fluence	$H_{irr}\left(T\right)$ shifts upward; improved $J_{c}\left(B\right)$ performance in high-field regime

**Table 4:** Summary of irradiation studies on REBCO and related superconductors.

Irradiation Type	Author (Year)	Sample	Irradiation Energy	Optimal Fluence	Improvement in $J_{c}/I_{c}$
Proton	Arya A. Soman et al. (2024) [46]	(Y, Dy)Ba_2_Cu_3_O_7−*δ*_	1.2 MeV/2.5 MeV	$1\times 10^{16}$ p/cm^2^/ $3\times 10^{16}$ p/cm^2^	At 20 K, 8 T, isotropic $I_{c}$ increased by ∼2.6×
Proton	Toshinori Ozaki et al. (2021) [19]	FeSe_0.5_Te_0.5_	1.5 MeV	$1\times 10^{16}$ p/cm^2^	At 5–10 K and <1 T, $J_{c}$ increased by ∼30%
Proton	Jia Y. et al. (2013) [106]	YBCO coated conductors	4 MeV	$8\times 10^{16}$ p/cm^2^	At 27 K, 6 T, $J_{c}$ increased by 1.8–2.0×
Heavy Ion	Gu et al. (2021) [107]	YBCO doped with Ta, Zr, Hf, Mn, Sn	1.9 GeV Ta ions	$5.0\times 10^{10}$ ions/cm^2^	At 30 K, 1 T, $J_{c}$ increased by 4.4×
Heavy Ion	A. Kujur et al. (2015) [108]	YBCO + 5 wt.% Y_2_O_3_	200 MeV Ag ions	$5\times 10^{12}$ ions/cm^2^	At 40 K, 0.04 T, $J_{c}$ increased by 2.48×
Heavy Ion	Martin W. Rupich et al. (2016) [109]	1.2 μm MOD YBCO (Dy_2_O_3_-doped)	16–18 MeV Au ions	$6\times 10^{11}$ ions/cm^2^	77 K self-field $I_{c}$ decreased by ∼35%; but increased >2× in 4–50 K and >1 T (H//c)
Neutron	M. Eisterer et al. (2024) [110]	Mixed fast (>0.1 MeV) and thermal (<0.55 eV) neutrons	Mixed spectrum	Low fluence $3.5\times 10^{16}$ m^−2^s^−1^; to high fluence $4.3\times 10^{22}$ m^−2^	Collision cascades enhanced pinning; maximum theoretical $I_{c}$ gain ∼30%
Neutron	D. X. Fischer et al. (2018) [104]	SuperPower GdBCO tapes	Thermal 29%, fast 36%	Low fluence $0.6\times 10^{22}$ to high fluence $3.9\times 10^{22}$ m^−2^	At 30 K, 15 T, $I_{c}$ increased at low fluence before saturation

**Table 5:** Comparison of different irradiation methods for REBCO coated conductors.

Irradiation Type	Dominant Energy Loss	Main Defect Types	Defect Scale & Morphology	Effect on $J_{c}$/$T_{c}$	Advantages	Limitations
Proton	Mainly NEL (minor EEL)	Point defects, small clusters	Random, nanoscale (1–5 nm)	Enhances $J_{c}$ at low *T* (<30 K), isotropic pinning; high fluence → $T_{c}$ drop	High controllability, low cost, uniform damage	Weak at high *T*; over-fluence induces disorder
Heavy Ion	Dominant EEL	Columnar defects, amorphous tracks	Continuous or discontinuous tracks (5–10 nm dia., 100–500 nm long)	Strong *c*-axis pinning; 2–3× $J_{c}$ gain under >5 T; slight $T_{c}$ reduction	Powerful correlated pinning; tunable directionality	High anisotropy; costly; local amorphization
Neutron	Elastic & inelastic collisions (NEL)	Cascade defects, clusters	Random isotropic defects (few nm), deep penetration	Moderate $J_{c}$ enhancement; isotropy improved; $T_{c}$ decreases with fluence	Deep uniform damage; wide *T*–*B* range	Poor control; radiation hazard; $T_{c}$ loss
Mixed (e.g., p + ion)	Combined EEL + NEL	Point + columnar + dislocation loops	Multiscale, partially correlated	Dual-region $J_{c}$ boost; isotropy improved; $H_{irr}$ up; stable creep	Synergistic pinning; wide applicability	Complex control; defect competition; reproducibility issues

## Data Availability

No new data were created or analyzed in this study. Data sharing is not applicable to this article.
